# Effectiveness of X-stop Interspinous Distractor Device Versus Laminectomy for Treatment of Lumbar Stenosis: A Systematic Review and Meta-Analysis

**DOI:** 10.7759/cureus.37535

**Published:** 2023-04-13

**Authors:** Chukwuyem Ekhator, Daniel Griepp, Alyssa Urbi, Brian Fiani

**Affiliations:** 1 Neuro-Oncology, New York Institute of Technology College of Osteopathic Medicine, Old Westbury, USA; 2 Neurosurgery, St. Barnabas Hospital Health System, Bronx, USA; 3 Neuroscience, Brandeis University, Waltham, USA; 4 Neurosurgery, Weill Cornell Medical Center/New York Presbyterian Hospital, New York, USA

**Keywords:** neurosurgery, interspinous distractor, laminectomy, x-stop, lumbar spinal stenosis (lss)

## Abstract

Lumbar spinal stenosis refers to the narrowing of the spinal canal in the lumbar region. There is an increasing need to determine the treatment modality for lumbar spinal stenosis by comparing the outcomes of X-stop interspinous distractors and laminectomy. The objective of this study is to determine the effectiveness of the X-stop interspinous distractor compared to laminectomy. This systematic review fundamentally abides by the procedures delineated in the Cochrane methodology while the reporting is done according to the Preferred Reporting Items for Systematic Review and Meta-Analyses guidelines. Three databases searched generated a total of 943 studies, with PubMed being the source for the bulk of the articles. Six studies were selected for inclusion in this study. The effectiveness of the interspinous distractor devices and laminectomy can be determined through their impact on the quality of life, rates of complications, and the amount of money utilized. This meta-analysis fundamentally emphasizes that laminectomy is a more effective intervention for the treatment of lumbar spinal stenosis as it is more cost-effective and results in fewer complications in the long term.

## Introduction and background

The spinal cord is a bundle of nerves that passes through the spinal canal formed by the vertebrae, and stenosis refers to the narrowing of this canal [1]. Therefore, lumbar stenosis refers to the narrowing of the spinal canal in the lumbar region. Stenosis of the spinal canal results in the compression of nerve roots that are located within the dural sac and foramina [2].

Typically, congenital conditions are responsible for idiopathic and achondroplastic lumbar spinal stenosis, while iatrogenic conditions are responsible for post-laminectomy and post-fusion stenosis [3]. Idiopathic stenosis arises from congenitally short pedicles including other morphologic features such as thick, squat pedicles, trefoil-shaped aspect of the canal, lateral recesses in the axial plane, and laterally directed laminae [4]. The primary cause of spinal stenosis is age-related degeneration, which commonly results in the constriction of the spinal canal due to various degenerative changes in the lumbar discs and facet joints; furthermore, excessive use of corticosteroids can also lead to spinal stenosis [5]. Constriction of the foramina is caused by declining disc height, expansion of annulus fibrosus, synovial cysts, expansion of the joint capsule, and facet osteoarthritis [6]. Lumbar spinal stenosis and intermittent neurogenic claudication share similar symptoms of pain and discomfort in the buttocks, thigh, and lower extremities during movement, with symptoms being exacerbated by lumbar extension and alleviated through flexion, while neurogenic claudication is a cluster of symptoms [7].

According to Katz et al., standing fundamentally involves extension which constricts the neural foramina and canal area causing impingement, whereas sitting down comprises flexion which expands the spinal canal, consequently mitigating impingement [8]. Physical examination of lumbar spinal stenosis shows wide-based gaits and instability which is caused by the proprioceptive fibers in the posterior columns [9]. Another major symptom of lumbar spinal stenosis is sensory and motor deficit. Typically, the deficit can be bilateral and mostly utilize more specific nerves. Lumbar spinal stenosis is fundamentally diagnosed in different ways [10]. During the diagnosis of lumbar spinal stenosis, healthcare providers look for signs of weakness, abnormal reflexes, and loss of sensation, and employ various tests such as X-rays, imaging tests, bone scans, myelograms, and electromyography to detect the condition [10,11].

Lumbar spinal stenosis, particularly when symptomatic, can be treated conservatively through pharmacological interventions, including medications, exercises, and physiotherapy strategies accompanied by pain management techniques. Typically, a large proportion of symptomatic patients do not experience change within the first year of the intervention [11]. Exercise is another conservative treatment for lumbar spinal stenosis and fundamentally involves strengthening the stomach, back, and leg muscles. Lumbar spinal stenosis can be treated through operative treatments which comprise the decompression of the spinal canal with laminectomies. Laminectomies can be accompanied by a partial facet atherectomy as well as an instrumented stabilization [12]. X-stop interspinous distraction device is increasingly becoming an attractive option for surgical techniques in dealing with lumbar spinal stenosis. Typically, the current healthcare system has been characterized by increasingly minimally invasive techniques during spine injuries of a deteriorated lumbar spine. According to Bagley et al., laminectomy is another surgical treatment for lumbar spinal stenosis and involves the removal of the bone spurs and tissues connected with the arthritis of the spine [13].

There is an increasing need to determine which treatment for lumbar spinal stenosis provides the most benefit by comparing the outcomes of X-stop interspinous distractors and laminectomy. The current body of literature strives to address the success and failures of both laminectomy and X-stop interspinous distractors without directly addressing the success rates of both [14]. Consequently, there is an overarching need to highlight the outcomes of competing treatments of lumbar spinal stenosis to improve the quality of care offered in clinical settings and to ensure that patients can make informed medical decisions in choosing a treatment plan for lumbar spinal stenosis. Lumbar spinal canal stenosis can arise from a wide range of congenital, iatrogenic, and acquired situations, the intricate interconnection between lumbar spinal stenosis and gait; hence, it is usually manifested through unsteadiness [15-18]. Some of the tests taken during the diagnosis of lumbar spinal stenosis include X-rays, imaging tests, and other studies including bone scans, myelograms, and electrical tests of muscle activity [19-22]. Deer et al. concluded that the competing treatments of symptomatic lumbar spinal stenosis include instrumented stabilization through X-stop interspinous distractors and surgical treatment embodied in laminectomy [20].

The primary objective of this literature review and meta-analysis is to assess the effectiveness of the X-stop interspinous distractor versus laminectomy in the treatment of lumbar spinal stenosis. The goal is to identify the treatment that offers the maximum benefit with fewer complications to the patient. The secondary objective is to compare the success rates of both treatments by evaluating the quality of life of patients after the interventions. The success of the interventions will be determined based on factors such as pain reduction, improvement in symptoms, mobility, and the likelihood of complications or disability. This meta-analysis will also focus on which treatment for lumbar spinal stenosis, particularly X-stop spinous distractors and laminectomy, is more effective by comparing their impacts on patients and their livelihoods.

## Review

Methodology

Design and Literature Search

The systematic review and meta-analysis fundamentally abides by the procedures delineated in the Cochrane methodology while the reporting is done according to the Preferred Reporting Items for Systematic Review and Meta-Analyses (PRISMA) guidelines. An all-encompassing electronic search was undertaken in the following databases: Cochrane Central Register, PubMed, and Google Scholar. Further relevant articles were obtained through the perusal of the reference lists of similar systematic reviews and meta-analyses. The study fundamentally included studies published by or after 2022. Typically, the search was further detailed through the use of Medical subject headings (MeSH) terms and a combination of keywords. The advanced search comprised the utilization of Boolean operators (AND/OR), field tags (tw and tiab), and truncations (Asterisks) to develop an all-compassing search string. The comprehensive search string comprised a combination and permutation of the keywords. ((effectiveness) OR (efficacy) AND (x-stop interspinous distractors) OR (celfax) AND (laminectomy) OR (unilateral laminectomy) OR (hemilaminectomy) OR (surgical decompression) AND (handling) OR (treatment) OR (dealing) AND (laminar spinal stenosis) OR (LSS) OR (spinal stenosis). The metanalysis also integrated several MeSH terms to make the search results more focused and relevant (“laminectomy” [Mesh] AND “X-stop interspinous distractor” [Mesh] AND “lumbar spinal stenosis” [Mesh]). The search string was used in the aforementioned databases to access relevant articles.

Eligibility Criteria and Data Extraction

The inclusion and exclusion strategy for the papers fundamentally relied on the PICO model which focuses on the participants, intervention, comparator, and outcomes. Therefore, papers included in this study focused on participants affected with lumbar spinal stenosis. Therefore, any other type of spinal stenosis or illness was omitted. Further, the model focused on the intervention, the X-stop interspinous distractor device; hence, any paper not focusing on the device was omitted. Only articles that addressed laminectomy were included in the study, and any other comparator was omitted. Finally, this meta-analysis focused on the outcomes of the laminectomy and X-stop interspinous distractor devices; hence, any articles that did not include the outcomes of these interventions were omitted. The main outcomes included the quality of life after intervention, complications, and the costs of the intervention. The effectiveness of the procedures was determined through their impact on the individual’s quality of life, the complications they experienced as a result of the treatment, and the expenses of the two treatments. Moreover, the meta-analysis primarily utilized the study design as an inclusion and exclusion strategy to be considered for inclusion. Therefore, only clinical trials and randomized controlled trials (RCTs). The currency of the articles also played a major role in the meta-analysis as it only included articles written in the last decade. Only articles that focused on human beings were included; hence, mammal and animal studies were excluded. Moreover, the meta-analysis only included studies that were written in the English language. Articles written in non-English languages were excluded. The data from the included articles were mined into a predesigned Microsoft Excel spreadsheet. This meta-analysis included the following: author (year of publication), study objective, participants, intervention, comparators, and outcomes. The outcomes of the studies were recorded, including the quality of life and the costs of the interventions. Any emerging incongruencies and variations were revised through deliberations and consultations with a third party.

Results

Study Selection Process

**Figure 1:**
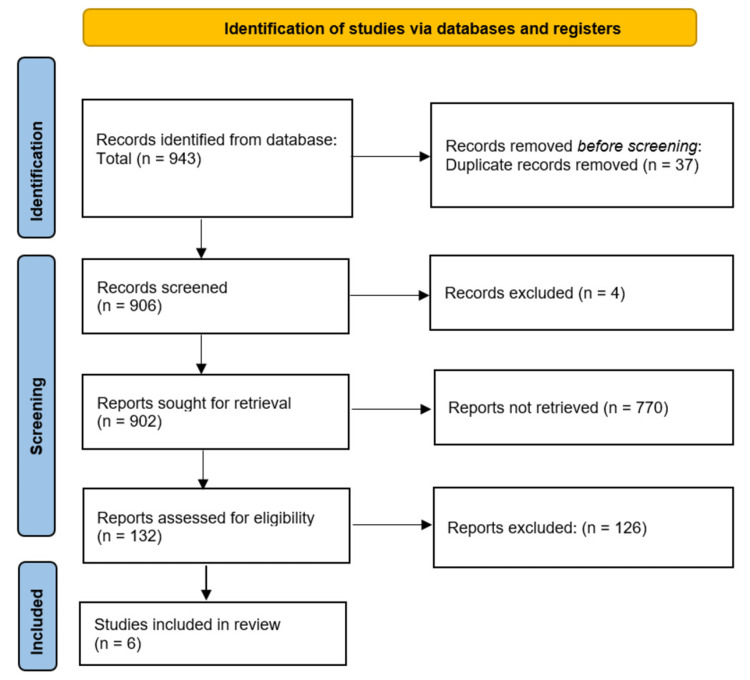
PRISMA flow diagram showing included studies. PRISMA: Preferred Reporting Items for Systematic Review and Meta-Analyses

Three databases searched generated a total of 943 studies in total, with PubMed being the source for the bulk of these articles. The breakdown of these search results is as follows: PubMed = 766, Google Scholar = 89, and Cochrane Central Library = 88. Duplicates (37) were first eliminated in the selection process, and four other studies were eliminated due to a lack of appropriate filing meta-data. A manual assessment of the four studies cemented their elimination as reasonable, and the remaining 902 studies were brought forth for the title and abstract screening. A total of 770 studies were eliminated due to ineligible objectives, lack of outcomes of interest, and ineligible topics or comparison of interest, among others. The remaining 132 articles were checked for methodological eligibility and appropriate reporting where 126 studies were eliminated. Six studies were selected for inclusion in this systematic review and meta-analysis. Figure 1 shows a PRISMA flow diagram representing the study selection process detailed above [23].

Table 1 shows a summary of characteristics extracted from the six selected studies.

**Table 1:** A summary of characteristics extracted from the six selected studies. IDD: internal disc compression; QALY: quality-adjusted life year; LSS: lumbar spinal stenosis; LL: lumbar laminectomy; IPD: interspinous process decompression

Author (year)	Study design	Study objective	Participants	Age (X-stop/laminectomy)	X-Stop	Laminectomy	Treatment success	Complications	Cost ($)	Quality of life	Conclusions
Borg et al. (2021) [24]	Multi-center, open-label, randomized controlled trial	The paper strives to establish the cost-effectiveness and long-term quality of life outcomes of X-stop IDD and laminectomy as treatments for LSS	47	70 (47–86)/69 (51–84) years	21	26	14 (66.67%) vs. 20 (76.92%)		Mean monetary cost ($5,408.59 vs. $2,849.28)	Mean QALY gain (0.81 vs. 0.92)	Laminectomy was more economical than the X-stop for treating LSS, primarily because of the price of the device. QOL did improve as a result of the X-stop device, albeit less so than in the laminectomy group
Borg et al. (2017) [25]	Randomized controlled trial	The article aims to establish the cost-effectiveness and quality of life following the treatment of LSS with the X-stop device or quality of life	47	70 years	21	26		2 (9.5%) vs. 5 (19.2%)	Mean monetary cost ($5,408.59 vs. $2,849.07)	Mean QALY gain (0.81 vs. 0.92)	For the treatment of LSS, laminectomy is more economical than X-stop implantation, mostly because of the price of the device. Laminectomy remains the gold standard of care
Patil et al. (2014) [26]	Retrospective comparative effectiveness study	The article aims to compare the reoperation rates, complication rates, and costs of interspinous devices and laminectomy	672	73 years	498	174	435 (87.4%) vs. 164 (94.2%)	17 (3.5%) vs. 16 (9.2%)	Cumulative costs ($39,173 vs $34,324)		Patients who received IDD compared to laminectomy for LSS experienced significantly higher 12-month reoperation rates and index hospitalization expenditures
Nurboja (2013) [27]	A multi-center randomized trial	The article strives to establish the cost-effectiveness of lumbar laminectomy against X-stop insertion for patients dealing with LSS	20	66 years	10	10		2 (20%) vs. 2 (20%)	Total cost in euros ($8,493.24 vs $5,779.95)	Mean QALY gain (0.593 vs 0.638)	In the NHS, lumbar laminectomy (LL) may be more affordable and cost-effective than X-stop over a 12-month period. No discernible differences between the two methods were found in terms of quality of life or clinical outcomes
Strömqvist et al. (2013) [28]	Randomized controlled trial	Comparing X-stop with conventional decompression in patients with neurogenic intermittent claudication due to LSS	100	67 (49–89)/71 (57–84) years	50	50	37 (74%) vs. 47 (94%)				Similar outcomes were obtained in both groups, although the X-stop group experienced more reoperations
Kondrashov et al. (2006) [29]	Comparative randomized prospective clinical study	To contrast laminectomy and IPD with the X-stop implant in patients with LSS in terms of clinical efficacy and direct hospital expenditures	30	68 years (SD 12.5)/69 years (SD 7.9)	18	12	14 (78%) vs. 4 (33%)	6 (33%) vs. 2 (17%)	Average direct hospital costs ($15,980 vs. $45,302).		At four years after surgery, IPD with the X-stop device for the treatment of LSS is clinically at least as effective as routine laminectomy and offers significant direct cost savings over decompressive surgery

Meta-analysis

Treatment Success

**Figure 2:**
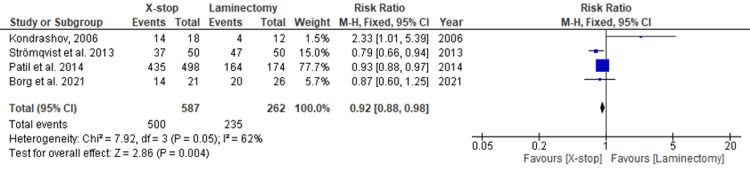
A forest plot of the comparison between X-stop and laminectomy assessing treatment success. Kondrashov et al. 2005 [29]; Stromqvist et al. [28]; Patil et al. 2014 [26]; Borg et al. 2021 [24].

**Figure 3:**
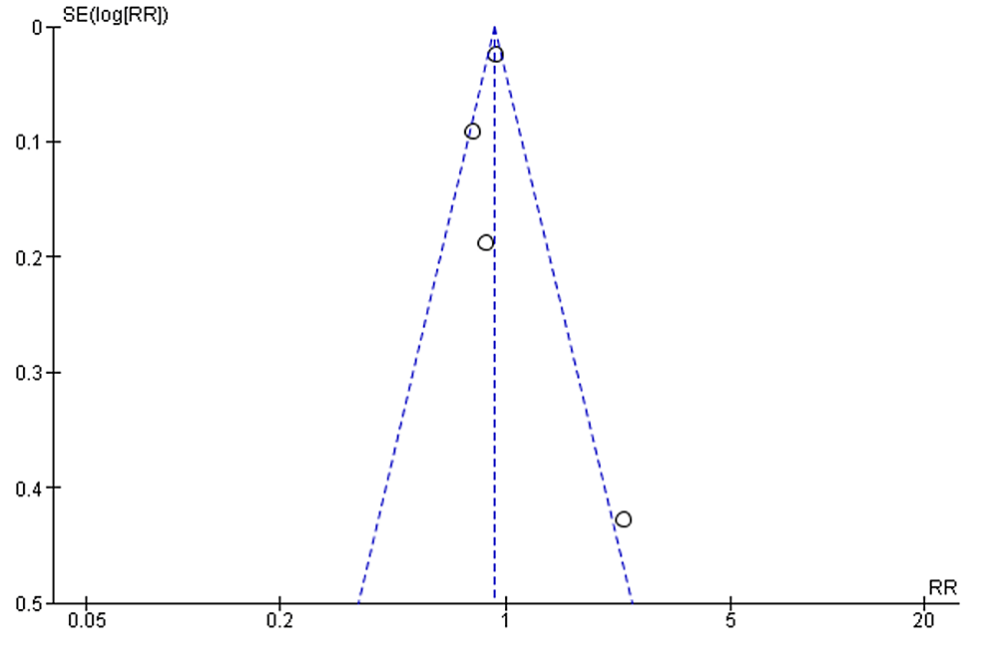
A funnel plot indicating publication bias between the included studies.

Four studies provide data for the following study outcomes. In total, 849 participants were included with 587 under X-stop and 262 under laminectomy. The frequency of treatment success was higher in the participants treated with laminectomy (90.08%) than in those treated with X-stop (85.18%). The meta-analysis found an effect measure of 0.92 (0.88, 0.98) fixed effects risk ratio at 95% CI. The heterogeneity between the studies was moderate (I^2^ = 62%). A significant difference in the success of treatment between X-stop and laminectomy was demonstrated (p = 0.004). Figure 2 and Figure 3 represent the forest and funnel plots of the meta-analysis.

Complications Identified in Included Studies

**Figure 4:**
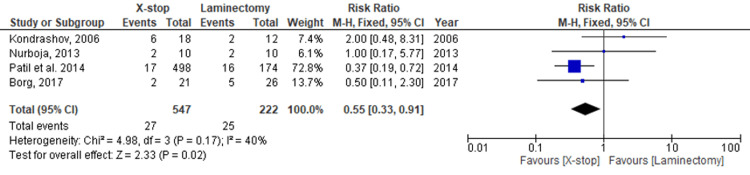
A forest plot of the comparison between X-stop and laminectomy assessing the incidence of complications. Kondrashov et al. 2005 [29]; Nurboja [27]; Patil et al. 2014 [26]; Borg et al. 2021 [24].

**Figure 5:**
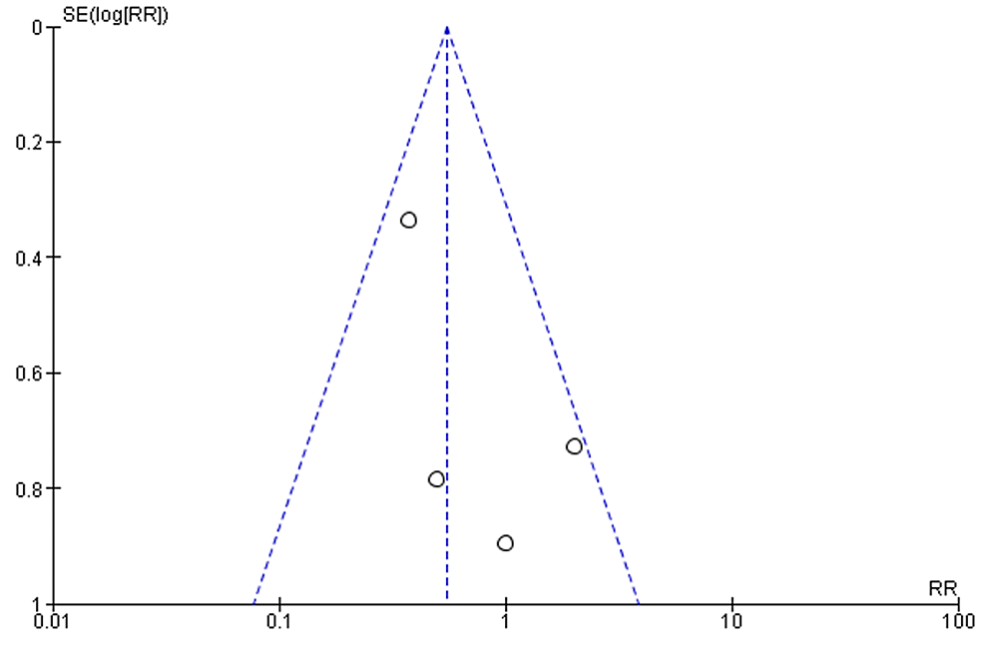
A funnel plot indicating publication bias between the included studies.

Four studies reported on the incidence of complications after the administration of these interventions. A total of 769 patients, randomized into X-stop (547) and laminectomy (222), were assessed. There was a higher complication rate in the laminectomy group (11.26%) than in the X-stop group (4.94%). The meta-analysis reports a fixed-effects risk ratio of 0.55 (0.33, 0.91) at 95% CI. The difference in complications between the two interventions is significant (p = 0.02). The outcome analysis presents a low level of heterogeneity (I^2^ = 40%). Figure 4 and Figure 5 represent the forest and funnel plots of the meta-analysis.

Discussion

With lumbar spinal stenosis increasingly gaining prevalence, there is an overarching need to determine the gold-standard treatment of the condition. Two of the competing treatments included surgical decompression through laminectomy and the placement of X-stop interspinous distractor devices. Nurboja identified that the effectiveness of the interspinous distractor devices and laminectomy can be determined through their impact on the quality of life, complication rates, and the amount of money utilized [27]. Therefore, the two treatments can be compared based on their effects on an individual’s economy and their general well-being. Patil et al. emphasized that laminectomy and X-stop interspinous distractor devices can be compared based on the number of reoperations required, which can also be associated with complication rates [26]. Generally, reoperations are made necessary due to complications or a negative effect on the quality of life. Borg determined that the efficacy of any treatment is established through the quality of life, cost, and complications [25].

Reoperations and Success of Treatment

Reoperation rates among laminectomy and X-stop interspinous distractor devices play a major role in determining their effectiveness. The interspinous distractor devices had a low reoperation rate in a short follow-up period, unlike laminectomy which had a high reoperation rate in that short follow-up period. According to Patil et al., after a follow-up period of over one year, the reoperation rate of interspinous distractor devices was significantly higher compared with laminectomy [26]. Therefore, interspinous distractor device and laminectomy both experience reoperations but the follow-up periods are different. The reoperation rates fundamentally affect the costs of the different treatments as well as indicating the quality of life.

Complications Rates Between X-stop Interspinous Device and Laminectomy

The meta-analysis establishes that laminectomy experienced higher complication rates than interspinous distractor devices due to the long stay in hospitals according to Patil et al. [26]. Generally, less invasive surgery is necessary to reduce complications and hospital stays. Borg determined that the complication rates for laminectomy were considerably higher [25]. The complications arising from interspinous distractor devices include spinous process fractures, device dislocations, and radicular deficits. The complications arising from interspinous distractor devices can be fundamentally corrected through better patient selection. Therefore, it is up to the patient to determine the best treatment depending on their condition. For instance, for patients with severe stenosis and foraminal stenosis, laminectomy is a better solution than an interspinous distractor device. Strömqvist et al. determined that after two years, X-stop interspinous distractor devices have relatively more complications than laminectomy [28].

Cost-Effectiveness of Care

Patil et al. determined that interspinous distractor devices were more costly than laminectomy due to the reoperation rates and the index hospitalization costs [26]. Generally, laminectomy was determined to be the most cost-effective strategy because of the high cost of X-stop interspinous distractor devices [25]. The devices are generally more expensive than any other clinical procedure. Nurboja emphasized that laminectomy has a lower cost than X-stop interspinous distractor devices [27]. The costs of laminectomy are due to the reoperation rates and quality of life. Laminectomy is a more cost-effective treatment for lumbar spinal stenosis [29].

Quality of Life

Katz and Harris emphasized that lumbar spinal stenosis has a profound effect on the patient’s well-being and general quality of life [1]. This meta-analysis fundamentally highlights that both the X-stop interspinous distractor devices and laminectomy had a positive effect on the quality of life. According to Borg, the treatment of lumbar spinal stenosis through X-stop interspinous distractor devices has the least improvement in the quality of life for patients [24]. Patil et al. determined that interspinous distractor devices have a higher rate of reoperations, highlighting that their effect on the quality of life is significantly lower creating a need for reoperations [26]

Study limitations

This study was limited by the small number of articles comparing the effectiveness of laminectomy and X-stop interspinous distractor devices. An insufficient number of publications comparing the articles fundamentally made it challenging to arrive at a definite conclusion about which of the two procedures is effective. The existence of other procedures and processes for treating laminar spinal stenosis such as minimally invasive decompression surgeries contribute to the limited number of cases. Further, during the meta-analysis, there was limited access to data as some of the articles which could have been included needed to be bought or required permissions from the authors. While the permissions could have been attained, the authors did not offer their permissions in time, consequently introducing time constraints.

## Conclusions

The meta-analysis fundamentally emphasizes that laminectomy is a more effective intervention for the treatment of lumbar spinal stenosis as it is more cost-effective and results in fewer complications over the long term. Generally, laminectomy is accompanied by complications over the short term due to postoperative care. The use of X-stop distractor devices is characterized by high costs as a result of the cost of equipment. Complications in the intervention of lumbar spinal stenosis using X-stop interspinous distractor devices are mainly due to the nature of the device and its alignment with the patient’s needs. Therefore, poor choice of device is the leading cause of complications in X-stop interspinous distractor devices interventions. Generally, x-stop devices are accompanied by a relatively higher rate of reoperations which significantly lowers their effectiveness. The insufficiency of publications addressing the effectiveness of lumbar spinal stenosis interventions essentially makes it challenging to conclusively determine that laminectomy is a more effective intervention than X-stop interspinous distractor devices. This study will help inform patients of lumbar spinal stenosis in determining which intervention to include in their treatment plans. Therefore, patients can easily determine which of the parameters of effectiveness is their priority making it easier for them to make informed decisions. This study is also crucial for clinicians as it raises awareness of the best alternatives they can offer to their patients when dealing with lumbar spinal stenosis.
